# The impact of behavioral weight management interventions on eating behavior traits in children with overweight or obesity: Systematic review and meta‐analysis

**DOI:** 10.1111/obr.13839

**Published:** 2024-09-19

**Authors:** Patricia Eustachio Colombo, Milindu Wickramarachchi, Aiswarya Lakshmi, Laura Kudlek, Amy Ahern, Struan Tait, Natasha Reid, Rebecca A. Jones, Andrea D. Smith

**Affiliations:** ^1^ School of Clinical Medicine University of Cambridge Cambridge UK; ^2^ Centre on Climate Change and Planetary Health London School of Hygiene and Tropical Medicine London UK; ^3^ MRC Epidemiology Unit, School of Clinical Medicine University of Cambridge Cambridge UK

**Keywords:** behavioral weight management, children, eating behavior traits, obesity treatment, systematic review

## Abstract

**Introduction:**

Behavioral weight management interventions (BWMIs) are an evidence‐based strategy for addressing childhood obesity. Targeting eating behavior traits (EBTs; individual tendencies determining food intake/occasions) could play a pivotal role in improving the effectiveness of these behavioral interventions. The present study describes a systematic review and meta‐analysis of the impact of BWMIs on eating behavior traits in children with overweight or obesity.

**Methods:**

Seven databases were searched, and eligible studies included randomized controlled trials reporting EBT outcomes following BWMIs delivered to children with overweight or obesity (<18 years of age). Random effects meta‐analyses were conducted to compare EBT outcomes for intervention and control groups. Synthesis without meta‐analysis (SWiM) was applied for EBTs where meta‐analysis was not feasible.

**Results:**

The review identified eight trials characterizing the impact of BWMIs on 15 EBTs. Meta‐analyses of data from three trials at intervention completion and post‐intervention (average of 28 weeks [±8]) revealed positive short‐term increases in dietary restraint (SMD random effect 0.42 [95% CI 0.13, 0.70]). However, these effects were not sustained at follow‐up. Improvements in emotional eating, external eating, food responsiveness, and enjoyment of food were shown in studies which could not be pooled quantitatively.

**Conclusion:**

BWMIs in children living with overweight/obesity are beneficial for the improvement of some EBTs at intervention completion including dietary restraint, emotional eating, external eating, food responsiveness, and enjoyment of food. However, this remains a relatively unexplored area and more research is needed to strengthen understanding of the multifaceted impact of child BWMIs on a comprehensive range of EBTs.

## 1 INTRODUCTION

In 2020, 175 million children aged 5–19 years were estimated to be living with obesity globally,^1^ and data show that obesity is increasingly developing earlier in life.^2^ Excess adiposity in childhood is associated with detrimental effects on metabolic and cardiovascular health outcomes, including type 2 diabetes, cardiovascular disease, and sleep apnoea.^3^ The impacts on children are not only physical, but also socio‐emotional, with obesity being described as “one of the most stigmatizing and least socially acceptable conditions in childhood”.^4^ Childhood obesity is challenging to reverse, tracks into adolescence and adulthood,^5^, ^6^ and the negative health effects follow into adulthood also.^3^ The high prevalence of childhood obesity and negative associated consequences highlights the need for effective weight management treatments.

Behavioral weight management interventions (BWMIs) addressing diet, physical activity, and lifestyle habits are key treatments for childhood and adolescent obesity. Reviews of behavioral interventions for the treatment of childhood obesity have reported positive effects for weight in the short and long term, with family based BWMIs prevailing as the main approach for supporting weight management in children and adolescents living with overweight or obesity.^7^ This is proposed to be due to parents and caregivers playing a key role in shaping children's energy‐balance related behaviours.^8^ As such, BWMIs are considered the first line of treatment for overweight and obesity in children and adolescents due to their effectiveness in promoting sustainable lifestyle changes. There continue to be important advances in pediatric obesity management with new treatment approaches including bariatric surgery and pharmacotherapy (e.g., GLP‐1 agonists).^9^ At present, guidelines only recommend pharmacotherapy for adolescents living with obesity as an adjunct to BWMI.^10^

Research suggests that variations in an individual's responses to food and food‐related cues, known as eating behavior traits (EBTs), could explain the variability in individual's responses to obesity treatments.^11^ Findings from a recent meta‐analysis of observational studies (*n* = 27 studies) demonstrate that EBTs which characterize a more avid appetite (e.g., food responsiveness) are associated with higher body mass index (BMI) z‐scores in children, while EBTs that capture tendencies which describes lower appetite (e.g., slowness in eating, satiety sensitivity) are linked with lower BMI z‐scores.^12^ Additionally, a latent class analysis identified distinct trajectories of EBTs were associated with different weight loss outcomes during family‐based treatment.^13^ This suggests that targeting different EBTs could play an important role in the effectiveness of BWMIs.

Previous studies have investigated the effect of BWMIs for children on weight, risk of eating disorders and other outcomes.^14^, ^15^, ^16^ However, previous reviews have not summarized the effects on a comprehensive range of EBTs. In addition, prior reviews have focused on specific types of interventions rather than including a broad array of intervention types. For example, Ash et al. specifically includes only BWMIs with an active family component, therefore missing out key interventions which likely are of relevance (e.g., school‐based interventions, healthcare‐based interventions, or peer‐led interventions).^17^ By including a wide range of intervention types in a synthesis of BWMIs on EBTs, we may conduct analyses to better understand whether a particular intervention type is more effective than another. Thus, more insightful implications and lessons for practice may be developed. The lack of systematic review of the impact of a varying types of BWMIs on a comprehensive range of EBTs creates a significant gap in knowledge, particularly given the important role EBTs are proposed to play in appetite regulation and weight management.^12^ Understanding these key behavioral factors and their utility in the context of weight management interventions is necessary to assess if and how to effectively target these traits to mitigate and manage childhood obesity.

In this systematic review with narrative synthesis and meta‐analysis, we assessed the impact of child BWMIs on EBTs compared to the usual care, inactive, or minimal intervention group. Furthermore, we map different intervention characteristics (delivery format, targeted behaviors, therapeutic approach, etc.) to identify whether specific types of interventions are more effective than others in changing EBTs.

## 2 METHODS

This systematic review and meta‐analysis followed the Preferred Reporting Items for Systematic Reviews and Meta‐Analyses (PRISMA) statement^18^ and was registered on PROSPERO (CRD42022367508).

### 2.1 Search strategy and selection criteria

A systematic search of the following seven electronic databases was conducted: *AMED*, *ASSIA*, *CINAHL*, *Cochrane database (CENTRAL)*, *Embase*, *MEDLINE*, and *PsycINFO*. Search terms were developed using combinations of relevant keywords and MeSH terms based on the following domains: (1) people living with overweight/obesity AND (2) weight management interventions AND (3) eating behavior trait outcomes AND (4) study designs. Searches were completed on September 29, 2022, and the search strategy is outlined on PROSPERO. Briefly, search results were imported into Mendeley software to remove duplicates. The remaining articles were transferred to Covidence systematic review software for screening. Articles were independently screened in duplicate, with any discrepancies discussed. Reference sections of relevant papers were also hand‐searched for further eligible studies.

Studies were included if they reported on a primary peer‐reviewed randomized controlled trial (RCT) or cluster RCT (cRCT) undertaken in children living in a community setting. Eligible participants were considered from 1–18 years of age, in line with the WHO definition of childhood.^19^ Children had to be living with overweight or obesity, defined as child adiposity ≥85th BMI weight centile or BMI > 25 kg/m^2^.^20^ Trials were included if they evaluated a behavioral weight management intervention. No restrictions were placed on intervention delivery (e.g., family‐based, parent‐based, social media) and duration. Interventions involving surgical and/or pharmacological intervention were excluded, as were interventions where participants were in‐patient. Trials had to include an inactive/wait‐list, minimal, or usual/standard care control group. Usual/standard care control group was evaluated on case‐by‐case basis in the context of each study to ascertain whether this control may include elements of a behavioral weight management program. The main outcome of interest in this review was EBTs. EBTs in this context were defined to capture any observable and measurable behavioral tendency of an individual which (i) shapes an individual's approach to initiate or cease an eating occasion and/or (ii) food intake in general (e.g., type, amount, frequency). An overview of EBTs and their desired direction of change in the context of a BWMI is shown in Supplementary Table S1. Given the age of eligible participants, EBT's were included if parent‐reported or self‐reported. Eligible trials were required to have reported at least one EBT at intervention end or any follow‐up. The EBT outcome had to be reported either as post‐intervention outcomes (SD) or change from baseline outcomes (SD).

### 2.2 Study selection and data extraction of included studies

Title, abstract, and full‐text screening was performed independently by two authors. Each record was screened by LK and duplicate screening was conducted by PEC, ST, NR, MW, and AL. Potential discrepancies were resolved through discussion with a third reviewer (RAJ). No automation tools were applied for the screening process.

Descriptive data on the study characteristics (setting, country, content), participant characteristics (*N* randomized, age, ethnicity, sex), EBTs measured, EBT tools/questionnaire, adiposity, and trial components (target behavior, delivery mode, delivery format, duration, frequency, intensity, intervention components and control group components) were extracted by two reviewers (PEC and AL/MW/RAJ/MS). Discrepancies were resolved through discussion with a third reviewer (LK or RAJ). When data for EBTs were not reported, corresponding authors (*N* = 5) were contacted and asked to provide data in the preferred format (mean with SD; baseline and at follow‐up). In cases where authors did not respond, a first reminding email was sent within 2 weeks and a second within 2 months of initial contact. Additional data were obtained from one study.^21^

### 2.3 Risk of bias assessment

**FIGURE 1:**
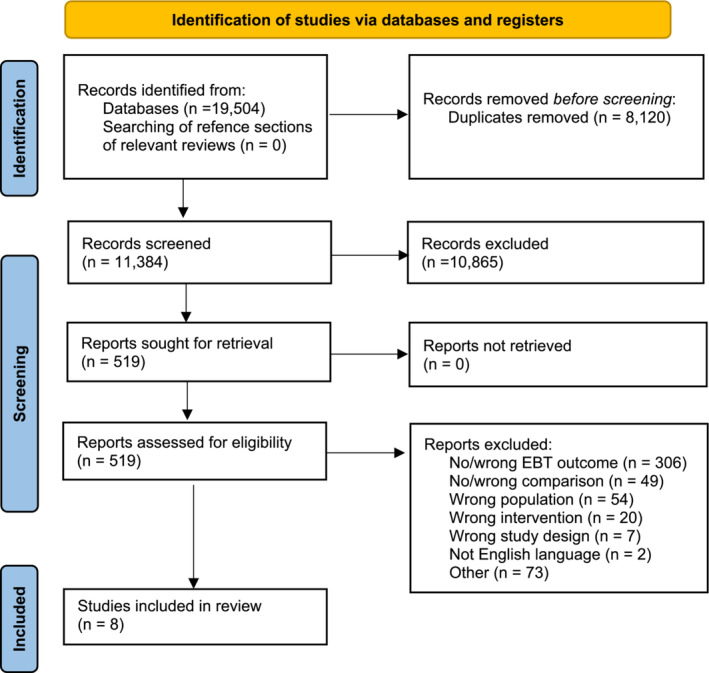
PRISMA flowchart of systematic literature review for inclusion of trials in this systematic review.

The risk of bias of each study was assessed using the Cochrane risk of bias tool for randomized trials (ROB 2.0).^22^ Two authors (PEC and AL/MW/RAJ) independently rated the studies. Discrepancies in scoring were resolved in discussion between PEC and the second author (AL/MW/RAJ/MS). In cases where essential characteristics of a study required for ROB 2.0 scoring were not sufficiently reported in the main eligible study, additional information was retrieved from the published study protocol. Funnel plots of individual trial effect sizes were produced for all outcomes to assess the potential for publication bias. Funnel plots for the meta‐analyzed studies were inspected visually (Figure 1; Figure S2).

### 2.4 Data synthesis for narrative review without meta‐analysis

Where there were too few studies reporting an EBT to pool them using meta‐analysis, a synthesis without meta‐analysis (SWiM) was applied.^23^ SWiM involves synthesizing and summarizing the findings from individual studies in a narrative or descriptive manner, rather than through statistical pooling of data, supporting transparency, and reproducibility in the synthesis process. Data from SWiM was graphically summarized using harvest plots.^24^ The method was originally developed to visualize effectiveness of public health interventions in scenarios where meta‐analysis is not applicable. The underlying principle of the harvest plot can be adapted to inspect a variety of study characteristics. In the present harvest plots, bar height indicates risk of bias, as scored using the ROB 2.0. The lower bar represents a study classed as “some concern,” with a higher bar representing “low concern” (lower risk of bias). Colors were added to indicating if the considered effectiveness indicator was obtained at intervention completion (dark gray) or at ensuing follow‐up (light patterned gray). Studies were further ordered according to reported intervention effectiveness, with studies classed as reporting a significant negative change, no change, or significant positive change in the eligible EBTs. Harvest plots were created in Microsoft PowerPoint (version 2023, Microsoft Corporation).

### 2.5 Data synthesis for meta‐analysis

The primary effect measure is the mean difference for a specific EBT from baseline to post‐intervention and follow‐up between intervention and the control groups. Where relevant data were presented with CIs instead of SDs, SDs were obtained by applying the formula indicated in the Cochrane handbook.^25^

### 2.6 Statistical analyses

All analyses were undertaken in R (version 4.30) using package “meta”^26^ for the meta‐analysis, creating inverse‐variance‐weighted forest plots to visualize the estimates.^27^ To combine EBT data outcomes across scales, data were pooled using the standardized mean differences (SMD). Intervention effects were estimated and combined across trials using random‐effects meta‐analysis at (a) end of intervention and (b) at 28 weeks (±8 weeks) from the end of intervention to investigate long‐term effects on EBTs. Between‐study heterogeneity was assessed with the *I* ^2^‐statistic and prediction intervals. Moderate between‐study heterogeneity was considered >50% for *I* ^2^ with levels of 75% deemed indicative of high inconsistency in approximation of the summarized effect size. As per Cochrane guidelines, post‐intervention outcomes and change from baseline outcomes were not combined in the same meta‐analysis/forest plot.^25^ Meta‐analyses were planned where ≥3 trials reported post‐intervention outcomes or change from baseline outcomes for a specific EBT. Subgroup analyses were planned to explore the influence of certain characteristics on observed pooled effect sizes. No sub‐group analyses were conducted due to low number of studies for meta‐analysis.

## 3 RESULTS

### 3.1 Literature search

A total of 19,504 articles were identified, of which 11,384 remained after removal of duplicates. Full text screening was completed for 519 articles. From these, a total of eight studies met eligibility for inclusion in the systematic review.^21^, ^28^, ^29^, ^30^, ^31^, ^32^, ^33^, ^34^ Figure 1 shows the number of papers identified in each stage of the review.

### 3.2 Study characteristics of included trials

Study characteristics are summarized in Table 1, with additional information in Supplementary Table S2. Six RCTs^28^, ^29^, ^30^, ^31^, ^33^ and two cluster RCTs^21^, ^32^ were identified. Sample sizes ranged from *n* = 44^30^ to *n* = 137.^33^ The geographical spread was mainly concentrated to high income countries in Europe (*n* = 3)^29^, ^33^, ^34^ and North America (*n* = 3),^28^, ^30^, ^31^ with only two trials from Asian middle‐income countries (Malaysia, Iran).^21^, ^32^ Overall, data on 15 different EBT outcomes were presented. The most frequently reported EBT was dietary restraint (*n* = 5), followed by external eating (*n* = 3), then food responsiveness (*n* = 2), emotional eating (*n* = 2), and satiety responsiveness (*n* = 2). Other EBTs were reported once, including disinhibition, negative affect eating, fatigue/boredom eating, eating related to hunger, emotional overeating, emotional undereating, desire to drink, enjoyment of food, slowness in eating, and food fussiness.

**TABLE 1:** Study characteristics of eligible management interventions on eating behaviors traits in children with overweight or obesity (*n* = 8).

			Participants			Trial				Outcomes			
Author (year) Trial name	Design	Country	*N*, gender (% F/M)	Age range/mean (SD)	Adiposity	Trial setting; delivery mode, format and target group	Comparison/theory	Intervention and control content	Duration and follow‐up	Target behaviors	EB questionnaires	EB trait	Effect on weight/adiposity
Ahmad (2020)^21^ REDUCE Intervention	cRCT	Malaysia	I: *n* = 67 (59.7% F, 40.3% M) C: *n* = 67 (56.7% F, 43.3% M)	I: 9.6 (1.2) years C: 9.6 (1.2) years	**BMI:** I:25.2 (3.5) kg/m^2^ C: 25.7 (3.9) kg/m^2^ **BMI‐z** I: 2.05 (0.40) kg/m^2^ C: 2.11 (0.39) kg/m^2^	Community Mixed delivery Both (individual and group) Family‐based intervention	Wait‐list control SCT	I: 16 weeks (4 weeks training + 12 weeks booster phase) Training phase: W1: 1 face‐to‐face session (parents only) W2 and W3: weekly Facebook sessions W4: 1 face sessions (parents and children) 12‐week booster phase Delivered via WhatsApp 22 h contact time. C: Wait‐list, receive the intervention 6 months after final data collection.	16 weeks Follow‐up: 12 weeks and 24 weeks post‐intervention	Eating behavior Dietary intake Physical activity Screen time Adiposity^46^	CEBQ	Enjoyment of food Satiety responsiveness Food responsiveness Emotional overeating^a^ Desire to drink^a^ Slowness in eating^a^ Emotional undereating^a^ Food fussiness^a^	Reported outcomes: **BMI‐z, WC%, and % BF** Adiposity data in Ahmad et al. (2018)^35^ **BMI‐z** Between‐group *p* = 0.05 **WC%** Between‐group *p* = 0.02 **%BF** Between‐group *p* = 0.05
Boutelle (2014)^30^ Regulation of Cues (ROC) program	RCT	USA	I: *n* = 22 (45.5% F, 54.5% M) C: *n* = 22 (54.5% F, 45.5% M)	I: 10.5 years (1.5) C: 9.9 years (1.10)	**BMI:** I: 28.0 (5.0) kg/m^2^ C: 26.5 (4.5) kg/m^2^	Clinical Face‐to‐face delivery Group delivery Family‐based intervention	Minimal SCT and BCTs	I: Simultaneous parent and child group sessions Content similar for children and parents, except that child‐specific materials were used (e.g., games). Weekly sessions for 12 weeks + biweekly for an additional 2 visits C: At‐home version of the program	16 weeks Follow‐up: 16 weeks post‐intervention	Appetite awareness	EAH‐PC scale CEBQ	External eating Negative affect eating Fatigue/boredom eating Food responsiveness Satiety responsiveness	Reported outcomes: **BMI, BMI‐Z, % OW** **BMI** Post‐treatment group × time *p* = 0.14 4 months follow‐up group × time *p* = 0.23 **BMI‐Z** Post‐treatment group × time *p* = 0.15 4 months follow‐up group × time *p* = 0.16 **%OW** Post‐treatment group × time *p* = 0.14 4 months follow‐up group × time *p* = 0.17
Doyle (2008)^28^ Student Bodies 2 (SB2)	RCT	USA	I: *n* = 40 (65% F, 35% M) C: *n* = 40 (60% F, 40% M)	I: 14.90 (1.70) years C: 14.10 (1.60) years Total: 14.50 (1.70) years	**BMI:** I: 34.8 (7.6) kg/m^2^ C: 33.6 (6.3) kg/m^2^	Community Remote delivery Both (individual and group) Family‐based intervention	Minimal CBT	I: Internet‐delivered multi‐component program (newsletter, journalling, discussion forum, calls) Discussion forums for adolescents Newsletter for parents with individualized feedback and activities + phone calls C: Usual care. Handouts on basic nutrition and physical activity info.	16 weeks Follow‐up: 16 weeks post‐intervention	Diet Physical activity Body image	EDE‐Q^36^	Restraint	Reported outcomes: **BMIz, BMI, weight (lb)** **BMIz** Post‐intervention between‐group *p* = 0.08 4 months follow‐up between‐group *p* = 0.02 **BMI** Post‐intervention between‐group *p* = 0.06 4 months follow‐up between‐group *p* = 0.03 **Weight (lbs)** Post‐intervention between‐group *p* = 0.03 4 months follow‐up between‐group *p* = 0.01
Jansen (2011)^29^ “Finger in the pie” CBT program	RCT	NL	I: *n* = 59 C: *n* = 39	Overall: 9.72 (1.60) years	**BMI %:** 96.48 (3.13) kg/m^2^	Community (health centers) Face‐to‐face delivery Group delivery Parent‐only intervention	Wait‐list CBT	I: In‐person groups (8 × 2 h educational sessions) C: Wait‐list control. Received intervention 6 months after final data collection	10 weeks Follow‐up: 12 weeks post‐intervention	Nutrition Physical activity Parenting skills	EDE‐Q^36^	Dietary restraint	Reported outcomes: **BMI %** **BMI %** Time × group interaction *p*‐value <0.01
Robertson (2017)^33^ “Families for Health” (FFH)	RCT	UK	I: *n* = 64 (58% F, 42% M) C: *n* = 73 (23% F, 20% M)	I: 9.46 (1.57) years C: 9.43 (1.61) years	BMI: I: 25.79 (4.44) kg/m^2^ C: 25.93 (4.32) kg/m^2^	Community Face‐to‐face delivery Group Family‐based intervention	Usual care Standard behavior change intervention	I: 10 weekly 2½‐hour sessions, with children and parents attending parallel groups. FFH included parenting skills, social and emotional development, healthy eating and physical activity. C: Usual care (Differed between regional centers, e.g., WeightWatchers)	10 weeks Follow‐up: 52 weeks post‐intervention	Physical activity Healthy eating	Family eating and activity habits questionnaire e (FEAHQ)	Eating related to hunger	Reported outcomes: **BMIz, %BF** **BMIz** 3 months post‐intervention between‐ group *p* = 0.63 12 months post‐intervention between‐ group *p* = 0.05 **%BF** 3 months post‐intervention between‐ group *p* = 0.52 12 months post‐intervention between‐ group *p* = 0.06
Saelens (2002)^31^ “Healthy Habits” program	RCT	USA	I: *n* = 23 C: *n* = 21 **Total** (41% F, 59% M)	Overall:14.2 (1.2) years	**BMI:** 30.7 (3.1) kg/m^2^	Clinical Mixed delivery Individual Family‐based intervention	Usual care Standard behavior change intervention	I: Multiple component behavioral weight control intervention Components included PC interaction and physician counselling in clinic + 4 months of telephone‐ and mail‐based behavioral counselling. C: Usual care. Individual and in person.	14–16 weeks Follow‐up: 16 weeks post‐intervention	Physical activity Nutrition	TFEQ	Dietary restraint Disinhibition	Reported outcome: **BMIz** **BMIz** Post‐treatment group × time *p*‐value = 0.02 3 months follow‐up group × time *p*‐value = 0.03
Salahshoornezhad (2022)^32^ Smart‐phone nutrition physical activity and CBT program	cRCT	Iran	I: *n* = 31 (100% F) C: *n* = 31 (100% F)	I: 10.5 (1.02) years C: 10.5 (1.02) years	**BMI:** I = 25.88 (2.60) kg/m^2^ C = 25.77 (2.98) kg/m^2^	School Mixed delivery Group Child‐only intervention	Usual care CBT	I: Multidisciplinary program consisting of weekly CBT, physical activity (3×/week), and a smart phone nutrition game (<45 min/day) C: Usual care. Routine healthy nutrition education for students and their parents.	10 weeks No follow‐up	Nutrition Psychological Physical activity	DEBQ	Emotional eating External eating Dietary restraint	Reported outcomes: **BMI, Weight (kg), WHR** **BMI** Between‐group change *p*‐value = 0.01 Reported outcomes: **Weight, WHR** **Weight (kg)** Between‐group change *p*‐value <0.001 **WHR** Between‐group change *p*‐value <0.87
Skjakodegard (2022)^34^ Family‐based behavioral social facilitation therapy	RCT	Norway	I: *n* = 59 (61% F, 39% M) C: *n* = 55 (56% F, 44% M)	12.6 (3.3) years C: 12.6 (2.8) years	**BMI:**31.9 (5.4) kg/m^2^ C: 31.7 (4.3) kg/m^2^	Outpatient clinic Face‐to‐face Individual Family‐based intervention	Usual care CBT	I: 17 sessions of structured CBT C: Usual care	I: 178 (47) days C: 374 (41) days No follow‐up	Diet quality Physical activity Sedentary behavior Sleep	DEBQ	Restrained eating External eating Emotional eating	Reported outcomes: **BMI‐SDS, %IOTF‐25** **BMI‐SDS:** Time × group interaction *p*‐value <0.001 **%IOTF‐25**: Time × group interaction *p*‐value <0.001

Abbreviations: %IOTF‐25, percentage above the International Obesity Taskforce threshold for overweight based on a child's age and sex; BCT, behavior change techniques; BL, baseline; BMI, body mass index; BMI‐SDS, body mass index standard deviation score; BMIz, BMI z‐score; C, control; CBT, cognitive behavioral therapy; CEBQ, Child Eating Behavior Questionnaire; cRCT, cluster randomized controlled trial; DEBQ, Dutch Eating Behavior Questionnaire; EAH, Eating in the Absence of Hunger; EDE‐Q, Eating Disorder Examination Questionnaire; F, female; I, intervention; M, male; RCT, randomized controlled trial; SCT, social cognitive theory; TFEQ, Three‐Factor Eating Questionnaire; WHR, wait‐to‐hip ratio.

^a^ Additional data obtained from corresponding author.

The average duration of interventions was 14.8 weeks (SD = 5.2), with the shortest interventions lasting 10 weeks^29^, ^32^, ^33^ and the longest intervention lasting 34 weeks.^34^ Two studies^31^, ^33^ were characterized as standard child behavioral interventions, including standard behavior change techniques for child weight management (e.g., suggesting joint family activities, providing educational booklets on health and parenting tips), nutrition counselling, etc. but did not employ structured BCTs to modify behaviors. In contrast, most (*n* = 6) were interventions based on either cognitive behavioral therapy (CBT) or social cognitive theory (SCT),^21^, ^28^, ^29^, ^30^, ^32^, ^34^ developed to modify behavior through strategies such as goal setting, self‐monitoring, and problem‐solving. Most interventions were delivered face‐to‐face only (*n* = 4)^29^, ^30^, ^33^, ^34^ or combined with remote elements (*n* = 3).^21^, ^31^, ^32^ One study was delivered solely remotely.^28^ Furthermore, interventions were delivered mostly in group (*n* = 4),^29^, ^30^, ^32^, ^33^ as individual sessions (*n* = 2),^31^, ^34^ or in a mixed format (*n* = 2).^21^, ^28^

### 3.3 Risk of bias assessment

From the eight included studies, most were deemed “low” risk of bias (*n* = 6/8), whereas two were classified as having “some” risk of bias^28^, ^29^ (Supplementary Table S2). Both studies received the “some concern” rating in domain 5: “Selection of the reported result” of the ROB 2.0 tool. Funnel plots were visually inspected and showed minor asymmetry (Supplementary Figures S1 and S2).

### 3.4 Effect of interventions on children's eating behavioral traits

Meta‐analyses were only possible for dietary restraint as a post‐intervention outcome. The remaining outcomes were analyzed narratively and visually using harvest plots.

### 3.5 Meta‐analysis of behavioral weight interventions on children's eating behavior traits

#### 3.5.1 Dietary restraint

**FIGURE 2:**
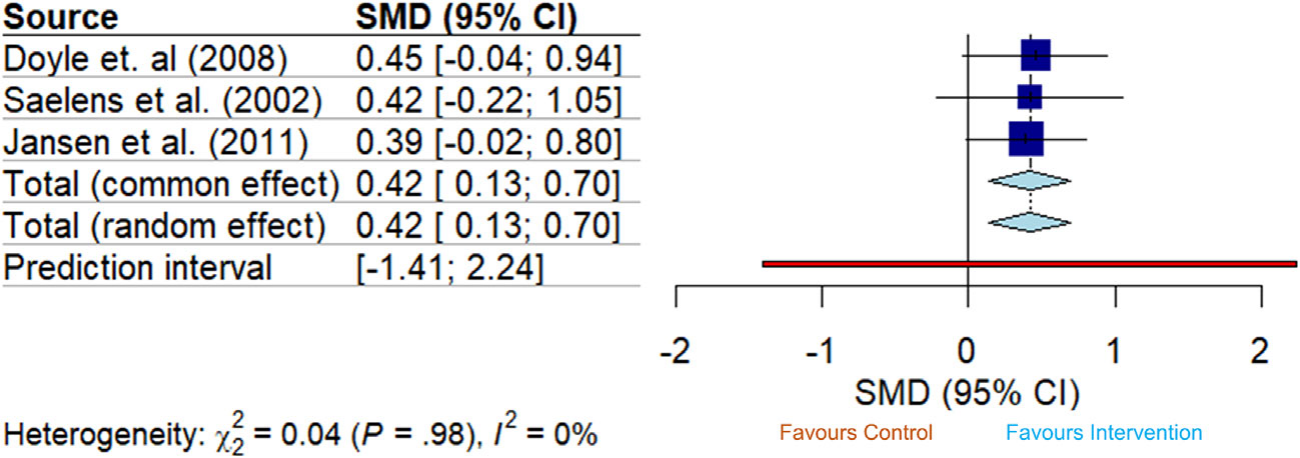
Forest plot showing the pooled intervention effect for dietary restraint where the desired effect is an increase in the trait at intervention end (*n* = 3). The size of blue squares representing each trial is proportional to the study weight. The width of the light blue diamond represents overall effect size corresponds to the length of the CI, and the red bar equates to the length of the prediction interval.

**FIGURE 3:**
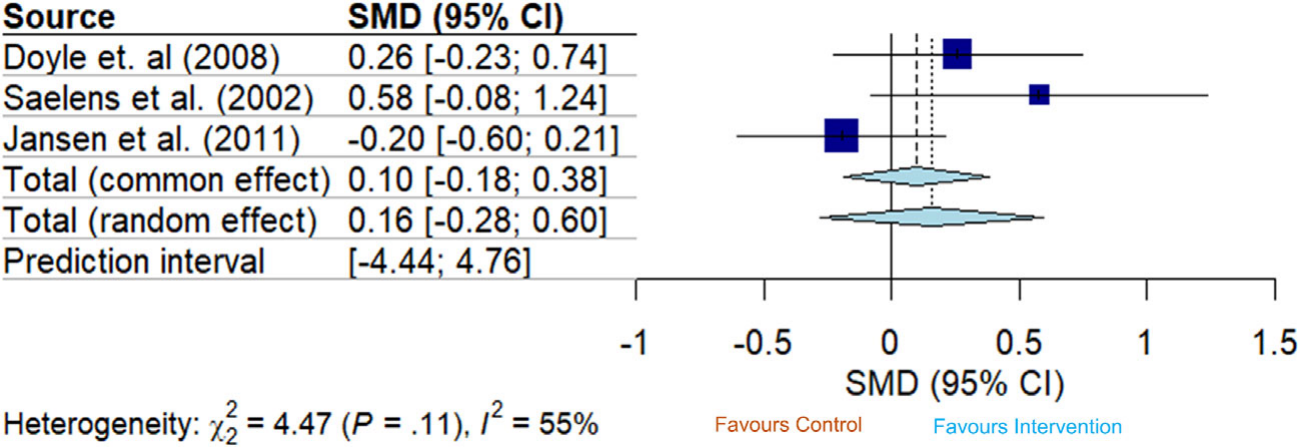
Forest plot showing the pooled intervention effect for dietary restraint where the desired effect is an increase in the trait at follow‐up (*n* = 3). The size of blue squares representing each trial is proportional to the study weight. The width of the light blue diamond represents overall effect size corresponds to the length of the CI. The prediction interval has been omitted as it spans beyond the limits of the displayed *x*‐axis.

Three trials reported post‐intervention outcomes for dietary restraint at intervention end. Two trials measured dietary restraint using the EDE‐Q,^36^ while one study by Saelens et al. (2022) used the TFEQ.^37^ When effect estimates were combined, there was evidence of a pooled effect of these interventions increasing dietary restraint at intervention completion (SMD random effect 0.42 [95% CI 0.13, 0.70, *I* ^2^ = 0%]) (Figure 2). The observed pooled effect diminished over time as there was no evidence of an effect on dietary restraint at follow‐up (SMD 0.16 [95% CI −0.28, 0.60, *I* ^2^ = 55%; Figure 3). Prediction intervals of pooled effect estimates crossed zero for both post‐intervention and follow‐up analyses (post‐intervention: [95% PI −0.41, 2.24]; follow‐up: [95% PI −4.44, 4.76]).

#### 3.5.2 Narrative review of behavioral weight interventions on children's eating behavior traits

**FIGURE 4:**
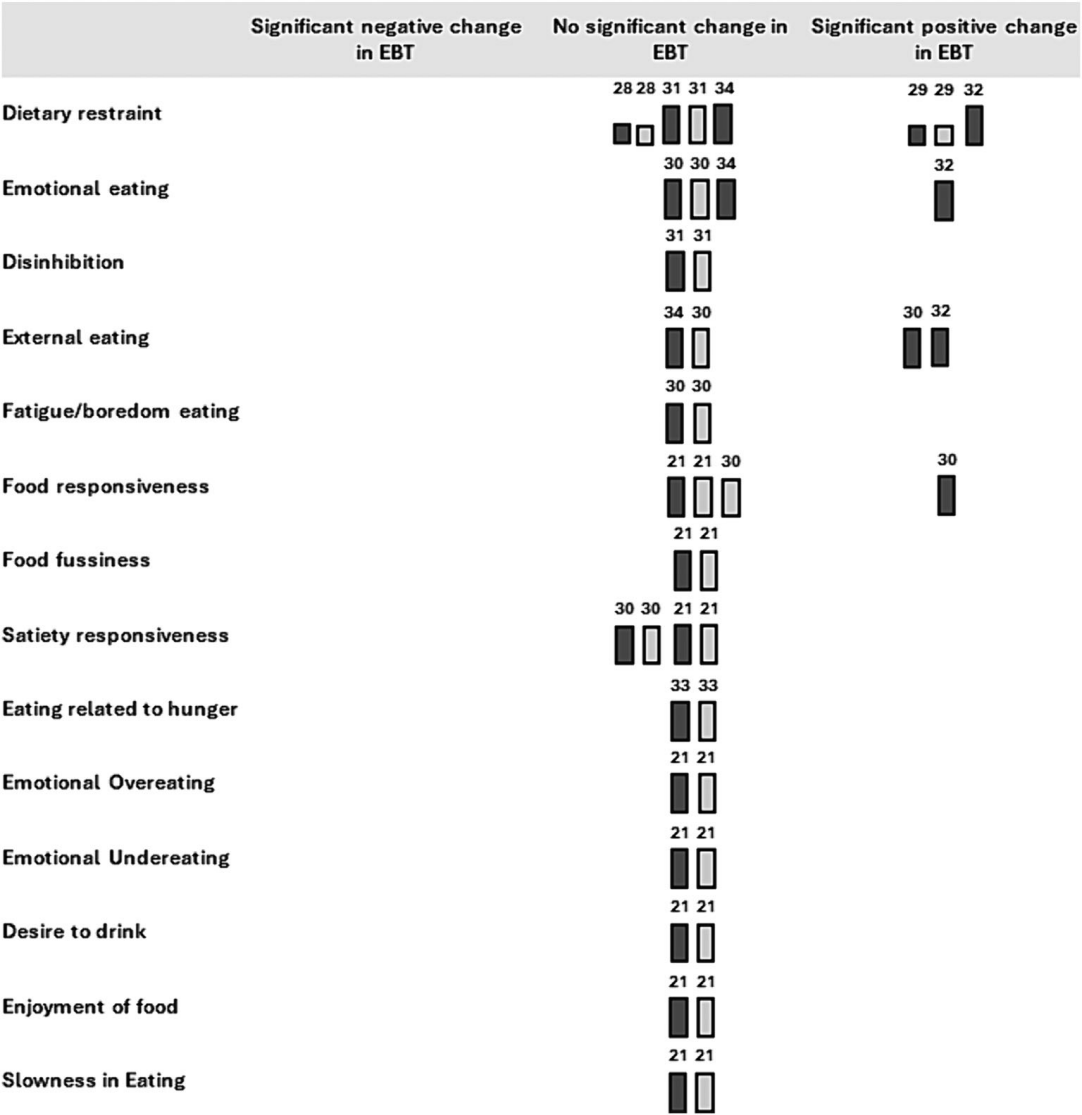
Harvest plot of intervention effects of eligible behavioral weight management interventions on eating behaviors traits in children with overweight or obesity (*n* = 8). Height indicating risk of bias (lower bar = some concern; higher bar = low); color indicating if it is a post‐treatment or follow‐up measurement (dark gray = post‐treatment measurement; light patterned gray = follow‐up measurement); number indicating the trial reference.

In the eight studies included in the systematic review, the impact of the interventions was reported for 14 different child‐EBT scores (positive, no effect, negative). Figure 4 summarizes the eight studies, including information on their risk of bias as ascertained using the ROB 2.0 tool.

#### 3.5.3 Dietary restraint

Two additional trials^32^, ^34^ reported change scores from baseline outcomes for dietary restraint; these data could not be included in the meta‐analytic models as per Cochrane guidelines^25^ (i.e., post‐intervention outcomes and change scores from baseline outcomes should not be combined in a meta‐analysis using SMD). In both these trials, dietary restraint was measured using the DEBQ.^38^ One trial found a significant positive effect of the intervention on the dietary restraint score at 10 weeks (*p* < 0.001)^32^ while the other found no evidence of a significant effect between‐ or within‐group differences for dietary restraint.^34^

#### 3.5.4 Emotional eating

Two studies reported children's emotional eating (EE)^32^, ^34^ measured using the DEBQ.^38^ One BWMI reported on negative affect eating^30^ ascertained using the Eating in the Absence of Hunger — parent‐report (EAH‐PC).^39^ Two studies reported the effect as a change score,^32^, ^34^ and one reported post‐intervention completion scores.^30^ The first study found a positive change in EE scores after 10 weeks of the multidisciplinary intervention relative to the standard nutritional education comparator group.^32^ The 6‐month program by Skjåkødegård et al. (2022) reported no changes in EE compared to the comparator group.^34^ For the study capturing negative affect eating in 4‐month appetite awareness–focused BWMI, the trial reported no significant intervention effect (*p* = 0.80) or at 4 months follow‐up (*p* = 0.65).^30^

#### 3.5.5 Disinhibition

Only one study reported on the impact on disinhibition — this was a 4‐month behavioral weight control program for adolescents with overweight. In the ‘Healthy Habits’ program, disinhibition was measured using the 51‐item version of TFEQ.^37^ The program was initiated in primary care with continuing telephone and mail contact and resulted in modest decreases in child BMI z‐scores. However, the intervention did not show a significant intervention effect on disinhibition.^31^

#### 3.5.6 External eating

Three studies investigated the impact of BWMIs on external eating.^30^, ^32^, ^34^ Two studies reported change scores in external eating measured using the DEBQ,^32^, ^34^ whereas one study measured external eating using the EAH‐PC^39^ at intervention completion and 4‐month follow‐up.^30^ The latter was the Regulation of Cues (ROC) intervention combining Children's Appetite Awareness Training with Cue Exposure Treatment Food interventions to influence children's external cues and internal hunger signals.^30^ This trial reported a significant positive effect (i.e., reduced score) on external eating *(p* < 0.05). However, this effect did not persist until the 4‐month follow‐up time point (*p =* 0.80). Likewise, the CBT‐based multidisciplinary study by Salahshoornezhad et al. (2022) found significant intervention effects on external eating when comparing baseline and post‐treatment scores (*p* < 0.001) after 10 weeks of the intervention in a sample of girls.^32^ The third study measuring external eating however did not find a significant intervention effect of the 6‐month family‐based behavioral social facilitation program on the EBT at intervention completion.^34^

#### 3.5.7 Food responsiveness

Two trials analyzed the effect of SCT‐based weight management interventions on children's food responsiveness. In both trials, food responsiveness was measured using the CEBQ^40^ and measured the effect between baseline and post‐intervention completion. The ROC program found a significant intervention effect (i.e., reduced score) on food responsiveness at intervention completion *(p* < 0.01), but not at 4 months follow‐up (*p* = 0.37).^30^ The second study, the family‐based intervention program (REDUCE), did not find a significant intervention effect on food responsiveness at intervention completion or follow‐up.^21^

#### 3.5.8 Satiety responsiveness

As with food responsiveness, two trials measured satiety responsiveness using the CEBQ.^40^ In both the ROC^30^ and REDUCE study,^21^ there was no significant intervention effect on satiety responsiveness at intervention completion or at follow‐up.

#### 3.5.9 Additional eating behavior traits

There were a few other EBTs which were only reported on in one singular study. These were the following: fatigue/boredom eating,^30^ eating related to hunger,^33^ emotional overeating,^21^ emotional undereating,^21^ desire to drink,^21^ enjoyment of food,^21^ slowness in eating,^21^ and food fussiness.^21^ The intervention effect was only significant for enjoyment of food in the REDUCE trial at intervention‐end (*p* < 0.01), 3 months follow‐up (*p* < 0.01), as well as 6 months follow‐up (*p* < 0.01).^21^

## 4 DISCUSSION

This systematic review and meta‐analysis identified eight trials that characterize the impact of BWMIs on 15 EBTs in children living with overweight or obesity, compared to no or minimal interventions or usual care. Results show that pediatric BWMIs resulted in positive short‐term effects on dietary restraint at program completion, in the small number of studies that measured this EBT. There was also evidence of interventions resulting in positive improvements in children's emotional eating, external eating, food responsiveness, and enjoyment of food. These improvements were predominantly observed at the end of the intervention, with effects no longer significant at later follow‐up. No evidence of differences between intervention and control were found for the remaining 10 EBTs (emotional overeating, boredom eating, eating related to hunger, disinhibition, satiety responsiveness, emotional undereating, desire to drink, slowness in eating and food fussiness). It is important to note that the very low number of contributing studies across outcomes mean generalizable conclusions are difficult to make. This review and meta‐analysis were not able to draw any further conclusions on the impact of these intervention on EBTS due to the low number of identified eligible studies to quantitatively pool findings.

The pooled estimates from our meta‐analysis of three trials found that, at program completion, BWMIs led to an increase in children's dietary restraint.^28^, ^29^, ^31^ This mirrors findings from previous systematic reviews and meta‐analyses,^41^, ^42^ showing an overall positive effect of BWMIs on dietary restraint in adults. In our analyses, the effect was not sustained at later follow‐up, suggesting that the increase in dietary restraint observed immediately after the interventions did not persist in the longer term. This contrasts findings from a recent systematic review and meta‐analysis in adults with overweight or obesity (Kudlek et al.; under review) that found evidence of increases in restraint following BWMIs, both at post‐treatment and 12 months follow‐up. The differences could potentially be explained by core developmental differences in adults and children. For children, dietary changes during BWMI are supervised by professionals, parents, and caregivers, and they may have less autonomy than adults in dietary choices made after the intervention ends. Thus, higher restraint during the intervention might reflect adherence to that diet rather than a change in trait dietary restraint. In the context of BWMIs, increased dietary restraint is often viewed as a means to achieve weight loss and improve weight management outcomes. It reflects the participant's ability to adhere to dietary guidelines and control food intake in response to palatable food, which are key components of successful weight management interventions. However, it is important to acknowledge that heightened dietary restraint can also be linked to an increased risk of developing disordered eating behaviours,^43^ particularly when it becomes excessive or rigid.^44^ So far, the worry over adolescent dieting and weight management has emerged from a number of observational studies but similar patterns are not mirrored in intervention studies.^14^ There are overarching trends in dietary restraint increasing across pediatric weight management interventions, but findings are still based on few data sources and reveal some inconsistencies. The results from our study indicate evidence of a pooled effect of BWMIs increasing dietary restraint at intervention completion, yet the effect diminished over time. When compared to the systematic review by House et al.,^15^ which examined changes in dietary restraint during pediatric weight management interventions with a dietary component and risk of eating disorders, their findings based on 20 studies reported that dietary restraint increased in most studies (*n* = 10) or remained unchanged. Taken together, this suggests that BWMIs can increase dietary restraint in young populations, with further research needed to understand the factors that contribute to varying outcomes. However, the low sustainability of effects may also be explained by the limited duration of the interventions. The range for children was 3–34 weeks, whereas in adults, this was 2–104 weeks (Kudlek et al.; under review). Sustainable, long‐term change may not be anticipated from short‐term, acute treatments.

The narrative findings further showed improvements in emotional eating, external eating, food responsiveness, and enjoyment of food among children that undertook BWMIs. Like dietary restraint, improvements were short term and not observed at follow‐up, except for enjoyment of food. Both our review and Jebeile et al.^14^ found improvements in emotional eating among children undergoing weight management interventions. This consistency suggests that BWMIs are promising as effective approaches in addressing emotional eating, which is a critical aspect of managing weight and promoting healthier eating behaviors in the long run. We found no evidence that the remaining 10 EBTs reviewed in this study were impacted by the BWMIs. However, we cannot draw reliable conclusions due to the limited number of studies measuring and reporting EBTs. Given the current mixed findings on the impact of BWMIs on EBTs in children living with overweight and obesity, it is important for future studies to measure and report on the traits to strengthen understanding in this area.

The choice of underlying theoretical framework may influence the mechanisms through which interventions impact EBTs. This review identified that interventions predominantly used approaches based on social cognitive theory (SCT) or cognitive behavioral therapy (CBT), with no studies utilizing third‐wave psychological therapy approaches (e.g., acceptance and commitment therapy). This approach is a more commonly accepted delivery model for weight management in adult populations. For instance, in a review and network analyses of 37 trials, the authors reported high‐quality evidence suggesting that third‐wave psychological therapy approaches yielded greater weight loss compared to no/minimal intervention control groups.^45^ The review also highlighted that differences between third‐wave psychological therapy approaches and other theory‐informed intervention were not consistent and effect sizes moderate. While promising, this area of BWMI requires extensive further research to establish which intervention delivery models are most effective, cost‐effective, and scalable. Importantly, it was not possible to test the effect of different intervention types due to the low number of contributing studies, but it would be valuable to unpick those differences in the future. More consistent measurement of EBTs would allow for such testing.

### 4.1 Strengths and limitations

This is the first systematic review that explores the impact of behavioral weight management approaches on different EBTs in childhood. Most of the studies were at low risk of bias. Two studies were considered to have some risk of bias and funnel plots show minor asymmetry which means that there was moderate publication bias. The ROB 2.0 domains that were scored as “some concerns” in the two studies include potential biases arising from the selection of the reported result. This typically means that the studies may have selectively reported outcomes based on the results, rather than following a pre‐specified analysis plan. To improve these studies, it is recommended that researchers in this field pre‐register their study protocols, ensure full transparency in reporting, and use comprehensive reporting guidelines (e.g., CONSORT for RCTs^46^). Publications in languages other than English or findings from gray literature were not included. Given the lack of standardized EBT tools and wide range of included traits, there was a high level of heterogeneity in the reporting of EBTs which made it challenging to synthesize the effects and draw reliable conclusions. The results are to be interpreted with caution given there was a small number of studies overall. The small number of studies eligible for meta‐analysis precluded the opportunity to apply meta‐regressions to quantify the importance of factors (child age, child sex, intervention program format) on observed effect.

### 4.2 Directions for future research

This review underscores a gap in research regarding the measurement and reporting of EBTs within the context of weight management interventions for children. Additional data will enable crucial analyses to understand the effect of intervention on EBTs and mechanisms of action influencing EBTs within BWMIs. Although behavioral interventions for the treatment of obesity in children may demonstrate a net positive effect for weight loss, these changes are unlikely to be experienced by all taking part in the intervention. In order to improve the effectiveness of these interventions for all, we must measure and better understand the influences on this individual variability. Additionally, longer interventions and follow‐up are needed to assess the long‐term effects of BWMIs. As seen with dietary restraint, some effects of BWMIs on EBTs may decline over time, and so one direction for future research is extending follow‐up timepoints for EBTs post‐intervention.

## 5 CONCLUSION

This systematic review and meta‐analysis of randomized controlled trials found that BWMIs are beneficial for the improvement of some EBTs at intervention end, including dietary restraint, emotional eating, external eating, food responsiveness, and enjoyment of food. Evidence supports improvements post‐treatment but not at longer‐term follow‐up. Our synthesis of findings and associated conclusions are limited by a low number of trials reporting EBTs in children. Our findings emphasize that this is a relatively unexplored area of research and more robust research is needed to strengthen our understanding. Future research would benefit from exploring novel intervention strategies leaning into advances in health technology, harmonies and routinely ascertain EBTs in weight management programs, and considering theory‐informed frameworks to advance the field of childhood obesity intervention and management.

## CONFLICT OF INTEREST STATEMENT

ADS, AL, LK, MW, NR, PEC, RAJ, and ST report no conflicts of interest. Since the completion of the systematic review, RAJ has commenced employment for WW (WeightWatchers). AA is on the Scientific Advisory Board for WW.

## RIGHTS RETENTION PILOT

For the purpose of Open Access, the author has applied a Creative Commons Attribution (CC BY) license to any Author Accepted Manuscript version arising.

## Supporting information


**Table S1.** Summary of eligible eating behaviours traits and their desired direction of change in the context of behavioural weight management interventions in children with overweight or obesity.
**Table S2.** Study characteristics of eligible behavioural weight management interventions on eating behaviours traits in children with overweight or obesity (*n*=9).
**Figure S1.** Funnel plot to assess publication bias for measurements of dietary restraint at intervention completion (*n*=3).
**Figure S2.** Funnel plot to assess publication bias for measurements of dietary restraint at follow‐up (*n*=3).
