# Supplementary material for: The impact of behavioral weight management interventions on eating behavior traits in children with overweight or obesity: Systematic review and meta‐analysis
Source: Obes Rev. 2024 Sep 19;26(1):e13839. doi: 10.1111/obr.13839 (PMC11611439; doi:10.1111/obr.13839)
Supplement: Supplementary file 1 — Table S1. Summary of eligible eating behaviours traits and their desired direction of change in the context of behavioural weight management interventions in children with overweight or obesity. Table S2. Study characteristics of eligible behavioural weight management interventions on eating behaviours traits in children with overweight or obesity (n=9). Figure S1. Funnel plot to assess publication bias for measurements of dietary restraint at intervention completion (n=3). Figure S2. Funnel plot to assess publication bias for measurements of dietary restraint at follow‐up (n=3). [file OBR-26-e13839-s001.pdf]

The impact of behavioural weight management interventions on eating behaviours traits in children with overweight or obesity: systematic review and meta-analysis

Supplementary materials

Patricia Eustachio Colombo[1,2*], Milindu Wickramarachchi [1*], Aiswarya Lakshmi [1*], Laura Kudlek[3], Amy Ahern[3], Struan Tait[3], Natasha Reid[1], Rebecca A. Jones [3**] Andrea D. Smith [3**]

[1] School of Clinical Medicine, University of Cambridge, Cambridge, United Kingdom

[2] Centre on Climate Change and Planetary Health, London School of Hygiene and Tropical Medicine, London, UK

[3] MRC Epidemiology Unit, School of Clinical Medicine, University of Cambridge, Cambridge, United Kingdom

Corresponding author: andrea.smith@mrc-epid.cam.ac.uk

Table S1. Summary of eligible eating behaviours traits and their desired direction of change in the context of behavioural weight management interventions in children with overweight or obesity

Eating behaviour trait	Desired change at intervention end
Dietary Restraint	Increase
Emotional eating	Decrease
Disinhibition	Decrease
External eating	Decrease
Fatigue/boredom eating	Decrease
Food responsiveness	Decrease
Satiety responsiveness	Increase
Eating related to hunger	Increase
Emotional Overeating	Decrease
Emotional Undereating	Decrease
Desire to drink	Unclear
Enjoyment of food	Unclear
Slowness in eating	Increase
Food fussiness	Decrease

Table S2. Study characteristics of eligible behavioural weight management interventions on eating behaviours traits in children with overweight or obesity (n=9)

Characteristics	Nr of Studies	Citations
**Study Design**		
RCT	6	[1–6]
Cluster RCT	2	[7,8]
**Overall risk of bias rating**		
Low	6	[1,4–8]
Some concerns	2	[2,3]
**Study location**		
USA	3	[2,4,7]
UK	1	[5]
Netherlands	1	[3]
Iran	1	[8]
Norway	1	[6]
Malaysia	1	[1]
**Sample Size (Total n randomized)**		
<100	5	[2–4,7,8]
100-300	3	[1,5,6]
**Delivery mode**		
Face to Face	4	[3,5–7]
Remote	1	[2]
Mixed	3	[1,4,8]
**Delivery format**		
Individual	2	[4,6]
Group	4	[3,5,7,8]
Both	2	[1,2]
**Intervention approach**		
Parent-only	1	[3]
Child-only	1	[8]
Family-based	6	[1,2,4–7]
**Intervention duration**		
<= 12 weeks	3	[3,5,8]
12-26 weeks	4	[1,2,4,7]
>=26 weeks	1	[6]
**Comparison type**		
Waitlist	2	[1,3]
Minimal	2	[2,7]
Usual Care	4	[4–6,8]
**Comparison intensity**		
No intervention[a]	5	[1–4,7]
Intervention[b]	3	[5,6,8]
**EBT outcomes reported at intervention end**		
Dietary Restraint	5	[2–4,6,8]
Emotional eating	2	[6,7]
Disinhibition	1	[4]
External eating	3	[6–8]
Fatigue/boredom eating	1	[7]
Food responsiveness	2	[1,7]
Satiety responsiveness	2	[1,7]
Eating related to hunger	1	[5]
Emotional Overeating	1	[1]
Emotional Undereating	1	[1]
Desire to drink	1	[1]

Enjoyment of food	1	[1]
Slowness in eating	1	[1]
Food fussiness	1	[1]
**EBT outcomes reported at follow-up**		
Dietary Restraint	3	[2–4]
Emotional eating	1	[7]
Disinhibition	1	[4]
External eating	1	[7]
Fatigue/boredom eating	1	[7]
Food responsiveness	2	[7,9]
Satiety responsiveness	2	[7,9]
Eating related to hunger	1	[5]
Emotional Overeating	1	[9]
Emotional Undereating	1	[9]
Desire to drink	1	[9]
Enjoyment of food	1	[9]
Slowness in eating	1	[9]
Food fussiness	1	[9]

[a]Includes one standardized email or one standardized provision of material)

[b]Includes at least one contact with some degree of personalization or several contacts)

Figure S1. Funnel plot to assess publication bias for measurements of dietary restraint at intervention completion (n=3)

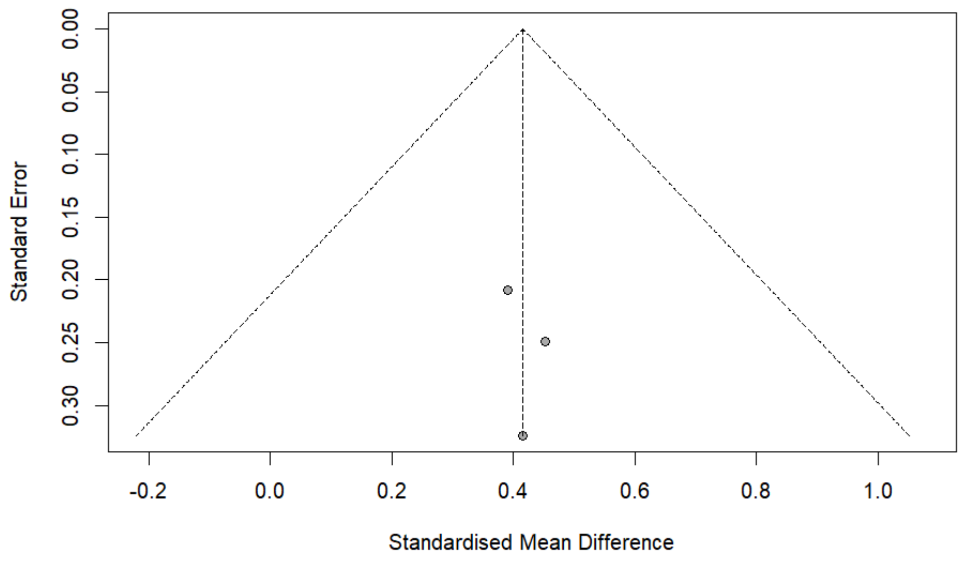

Figure S2. Funnel plot to assess publication bias for measurements of dietary restraint at follow-up (n=3)

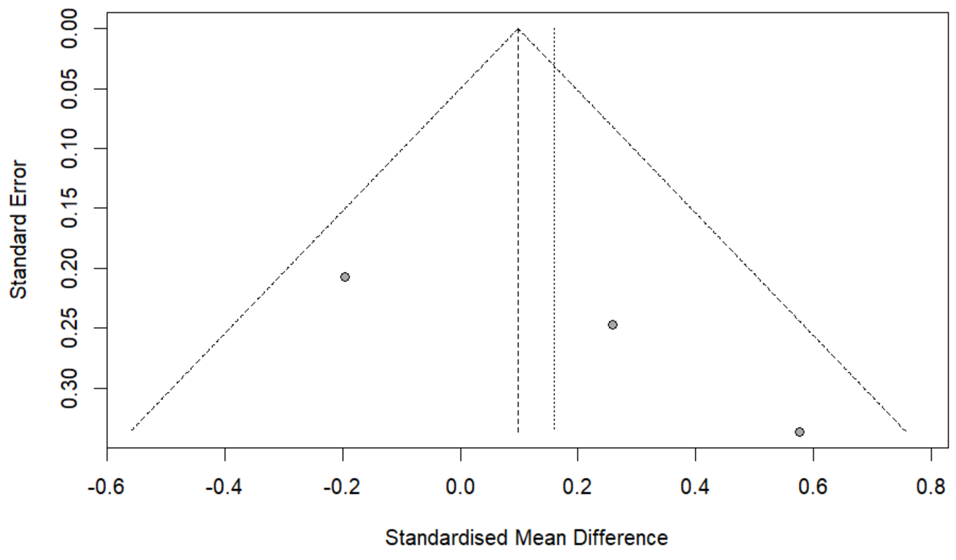

References

1. Ahmad, N., Shariff, Z. M., Mukhtar, F. & Lye, M.-S. Effect of Family-Based REDUCE Intervention Program on Children Eating Behavior and Dietary Intake: Randomized Controlled Field Trial. *Nutrients* **12**, 3065 (2020).
2. Doyle, A. C. *et al.* Reduction of overweight and eating disorder symptoms via the Internet in adolescents: a randomized controlled trial. *J Adolesc Health* **43**, 172–179 (2008).
3. Jansen, E., Mulkens, S. & Jansen, A. Tackling childhood overweight: treating parents exclusively is effective. *Int J Obes (Lond)* **35**, 501–509 (2011).
4. Saelens, B. E. *et al.* Behavioral Weight Control for Overweight Adolescents Initiated in Primary Care. *Obesity Research* **10**, 22–32 (2002).
5. Robertson, W. *et al.* Randomised controlled trial and economic evaluation of the 'Families for Health' programme to reduce obesity in children. *Archives of Disease in Childhood* **102**, 416–426 (2017).
6. Skjåkødegård, H. F. *et al.* Family-based treatment of children with severe obesity in a public healthcare setting: Results from a randomized controlled trial. *Clinical Obesity* **12**, e12513 (2022).
7. Boutelle, K. N. *et al.* An Intervention Based on Schachter's Externality Theory for Overweight Children: The Regulation of Cues Pilot. *Journal of Pediatric Psychology* **39**, 405–417 (2014).
8. Salahshoornezhad, S. *et al.* Effect of a multi-disciplinary program on anthropometric and biochemical parameters in obese and overweight elementary school girls: A randomized clinical trial. *Nutrition, Metabolism and Cardiovascular Diseases* **32**, 1982–1989 (2022).
9. Ahmad, N., Shariff, Z. M., Mukhtar, F. & Lye, M.-S. Family-based intervention using face-to-face sessions and social media to improve Malay primary school children's adiposity: a randomized controlled field trial of the Malaysian REDUCE programme. *Nutr J* **17**, 74 (2018).
